# Dried Wild-Grown Mushrooms Can Be Considered a Source of Selected Minerals

**DOI:** 10.3390/nu14132750

**Published:** 2022-07-01

**Authors:** Karolina Orywal, Katarzyna Socha, Patryk Nowakowski, Wojciech Zoń, Barbara Mroczko, Maciej Perkowski

**Affiliations:** 1 Department of Biochemical Diagnostics, Faculty of Pharmacy with the Division of Laboratory Medicine, Medical University of Bialystok, Waszyngtona 15A, 15-269 Bialystok, Poland; barbara.mroczko@umb.edu.pl; 2 Department of Bromatology, Faculty of Pharmacy with the Division of Laboratory Medicine, Medical University of Bialystok, Mickiewicza 2D, 15-222 Bialystok, Poland; katarzyna.socha@umb.edu.pl (K.S.); patryk.nowakowski@umb.edu.pl (P.N.); 3 Department of Public International Law and European Law, Faculty of Law, University of Bialystok, Mickiewicza 1, 15-213 Bialystok, Poland; w.zon@uwb.edu.pl (W.Z.); m.perkowski@uwb.edu.pl (M.P.)

**Keywords:** dried mushrooms, calcium, magnesium, iron, zinc, copper, manganese, selenium, atomic absorption spectrometry, nutrition declaration

## Abstract

Dried mushrooms might be a source of mineral components, which are indispensable for human health. The aim of this study was to determine the contents of calcium (Ca), magnesium (Mg), iron (Fe), zinc (Zn), copper (Cu), manganese (Mn), and selenium (Se) in dried wild-grown mushrooms (*Boletus edulis* and *Xerocomus badius*) available for sale, and to evaluate these mushrooms’ contribution to the daily reference intake of the studied bioelements. The concentrations of mineral components in the mushroom samples were determined by the flame method (Ca, Mg, Fe, Zn, Cu, Mn) and the electrothermal (Se) atomic absorption spectrometry method. The mean Ca, Mg, Fe, Zn, Cu, Mn (in mg/kg), and Se concentrations (in µg/kg) in *B. edulis* were 82.1, 964.1, 233.4, 97.9, 25.3, 22.1, and 6501.6, respectively, whereas in *X. badius*: 67.5, 1060.2, 87.8, 197.2, 33.9, 19.8, and 282.4, respectively. We have shown that dried *B. edulis* can be considered a source of Se. In the case of the other microelements, the tested mushrooms may serve only as additional supplements. Therefore, the studied species of mushrooms cannot be regarded as potential nutritional sources of the macroelements in question. Consumers should be properly informed about this, which should be guaranteed by appropriate legal regulations.

## 1. Introduction

**Figure 1:**
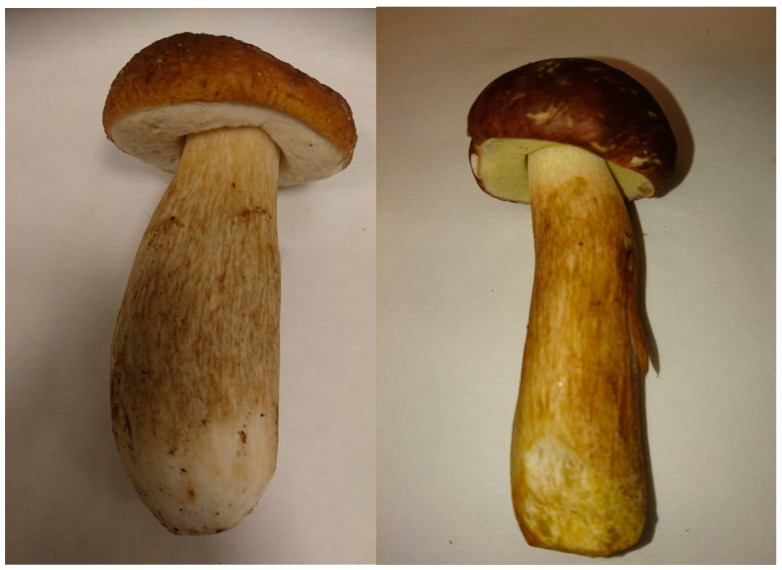
*Boletus edulis* (**left**) and *Xerocomus badius* (**right**).

The tradition of collecting mushrooms is widespread throughout Poland, one of the leading suppliers of wild-growing mushrooms in Europe, mostly King Bolete (*Boletus edulis* Bull., 1782) [1]. Only in 2019, 847 tons of King Bolete were exported to countries in Europe: Lithuania (317 tons), Germany (187 tons), Italy (142 tons), Romania (71 tons), France (38 tons), Switzerland (38 tons), and Portugal (22 tons) [2]. Drying is the most common method of mushroom processing and dried mushrooms are mostly used for home consumption or food service, also as an ingredient in the food industry: as a spice mix or in dehydrated soups. The most popular species of mushrooms sold in the dried form are *B. edulis* (En. penny bun, Es. boleto comestible, De. steinpilze, Fr. cèpe de Bordeaux, It. porcini) and *Xerocomus badius* (En. brown bay bolete, Es. bolete de bahía marrón, De. maronenpilz, Fr. bolet baie, It. porcini di alloro marrone) (Figure 1). Dried wild-growing mushrooms from Poland are commercially available in supermarket chains all over Europe and are considered to be a functional food, thanks to their content of physiologically active ingredients which provide health benefits. Most edible mushrooms are an important source of nutrition, thus wild-growing species are gaining steady popularity as commercial products, increasingly appreciated as a delicacy. Mushrooms are a good source of digestible proteins and fiber, are low in fat and energy, and offer a beneficial combination of vitamins and minerals in harmonious proportion [3].

Minerals necessary for the human body include: calcium, magnesium, iron, zinc, copper, manganese, and selenium. One of the most important functions of calcium (Ca) is skeleton mineralization. This element is required for normal growth, development, and bone strength. Furthermore, Ca participates in mediating muscle and vascular contraction and vasodilatation, nerve impulse transmission, and is the fourth coagulation factor [4]. Ca deficiency can result in rickets in children and osteomalacia, and increased risk of osteoporosis in adults. Low Ca is also associated with preeclampsia and hypertension [5]. Magnesium (Mg) is involved in over 600 enzymatic reactions, including all reactions that implicate ATP, protein, and nucleic acid synthesis; it participates in immune response, and plays an important physiological role, particularly in the brain, heart, and skeletal muscles [6]. Mg deficiency can be associated with increased production of oxygen-derived free radicals and systemic inflammation related to increased levels of proinflammatory molecules, such as IL-6, TNF α, and fibrinogen [7,8]. Iron (Fe), as a component of heme, is responsible for oxygen transport; it also participates in cellular energy metabolism, and is a cofactor in many enzymatic reactions. Fe deficiency impairs organ function and causes anemia, which is the most common nutritional disorder affecting a quarter of the world population [9]. Zinc (Zn) is essential to the human body because of its role in multiple biological processes in human cells. It is a cofactor of many enzymatic reactions and, by binding to proteins in the human proteome, it maintains its structural integrity. This ion can also directly regulate kinases, phosphatases, or channel activities [10]. Moreover, Zn exhibits anti-oxidative and anti-inflammatory properties; its deficiency leads to growth disorders, anemia, neuronal dysfunctions, as well as cardiovascular diseases [11]. What is more, Zn deficiency is involved in COVID-19 due to inhibition of RNA-dependent RNA polymerase, which blocks viral replication in coronavirus respiratory tract infections [12]. Similar to Zn, copper (Cu) is a cofactor of many redox enzymes, such as ceruloplasmin and CuZn-superoxide dismutase. Cu is required for normal immune response, collagen and elastin synthesis, bone formation, iron and heme metabolism, antioxidant defense, proper functioning of the nervous system, and neuropeptide synthesis [13,14]. A deficit of Cu in infancy may result in impaired development of the cardiovascular system, bone malformation, as well as neurologic and immunologic abnormalities [15]. In adults, Cu deficiency has been associated with alterations in cholesterol metabolism and immune malfunction, most pronounced in older patients [16,17]. Manganese (Mn) takes part in the metabolism of proteins, lipids, and carbohydrates. It acts as a cofactor for numerous enzymes, also involved in effective defense against reactive oxygen species. In addition to that, Mn is needed for collagen synthesis, as a cofactor in the collagen cross-linking process [18]. Mn deficiency is rare but insufficient Mn levels have been shown to adversely affect growth, bone formation, glucose tolerance, as well as lipid and carbohydrate metabolism [18,19]. Selenium (Se) is an essential micronutrient, because of its incorporation into selenoproteins—enzymes crucial to human health since they modulate cardiovascular, antioxidant, immune, metabolic, and thyroid functions [20]. Se deficit may result in cardiac, muscular, osseous, and immune disturbances [21]. Moreover, Se deficiency is an established risk factor for viral infections, including COVID-19, and mortality risk from severe diseases, e.g., sepsis or polytraumatic injury [22,23,24]. Minerals are supplied to the human body with food, and mushrooms have a strong capacity to absorb trace elements from the soil. An appropriate composition and concentration of microelements can be beneficial to humans, preventing cancer, hypercholesterolemia, diabetes, hypertension, inflammation, liver dysfunction, viral and bacterial infections, or cardiovascular diseases [25,26,27].

Based on these aforementioned arguments, estimation of the content of trace elements in food products is important in the context of public health. The aim of this study was to determine the concentrations of Ca, Mg, Fe, Zn, Cu, Mn, and Se in dried wild-grown mushrooms (*B. edulis* and *X. badius*) available for sale, and evaluate mushrooms’ contribution to the daily intake of the analyzed bioelements. Additionally, the aim of this study was to verify whether dried mushrooms could be regarded as a source of essential minerals in the diet of the adult population. 

## 2. Materials and Methods

### 2.1. Materials

The studied material consisted of 80 samples of dried edible mushrooms from two species most frequently consumed in Poland: *B. edulis* (40 samples) and *X. badius* (40 samples). The mushrooms were purchased in supermarkets belonging to 5 different European chains, and according to the producers, they had been harvested in 2019–2020. All purchased dried mushrooms had been produced by Polish companies, and Poland was indicated as the country of origin of the mushrooms. The characteristics of the studied samples are presented in Table 1.

### 2.2. Sample Preparation Procedure

The mushroom samples were powdered using a mechanical homogenizer (IKA Ultra-Turrax T18 digital, Staufen, Germany) and stored in polypropylene containers at −20 °C until analysis. To determine the concentrations of Ca, Mg, Fe, Zn, Cu, Mn, and Se, powdered mushrooms (0.2–0.3 g weighed with accuracy 1 mg) were mineralized with concentrated (69%) nitric acid (Tracepur, Merck, Darmstadt, Germany) using a closed-loop microwave system (Speedwave, Berghof, Eningen, Germany). The mineralization process consisted of four steps with different temperatures (1st—170 °C, 2nd—190 °C, 3rd—210 °C, 4th—50 °C) and pressure (1st—20 atm, 2nd—30 atm, 3rd—40 atm, 4th—40 atm), and lasted for 10, 10, 10, and 18 min, respectively.

### 2.3. Determination of Concentrations of Mineral Components

The concentrations of mineral components in the mushroom samples were determined by the acetylene-air flame method (Ca, Mg, Fe, Zn, Cu, Mn) and the electrothermal (Se) atomic absorption spectrometry method with the Zeeman background correction (Z-2000 instrument, Hitachi, Tokyo, Japan). The procedure was performed according to the manufacturer’s recommendations. To determine the content of Se, a palladium–magnesium matrix modifier (Merck, Darmstadt, Germany) was added (Pd concentration: 1500 mg/L; Mg concentration: 900 mg/L). In the case of Ca and Mg, 1% lanthanum chloride (LaCl3, Sigma-Aldrich, Merck, Darmstadt, Germany) was adopted as a masking agent. The concentrations of Ca, Mg, Fe, Zn, Cu, and Mn in the samples were presented as mg/kg of dried mushrooms, whereas the concentration of Se as μg/kg of dried mushrooms. Before the analysis, the majority of the mineralized samples were diluted 10 times for Ca, 50 for Mg, 5 for Fe, 20 for Zn, 5 for Cu, 10 for Mn, and 2 for Se. The analytical conditions of the processes involved in the flameless and flame techniques are presented in Table 2.

### 2.4. Accuracy Check of the Methods

Quality control was performed by analyzing certified reference materials, CS-M-3 Dried Mushroom Powder (Institute of Nuclear Chemistry and Technology, Warsaw, Poland) and Simulated diet D (Livsmedels Verket National Food Administration, Sweden). The certified materials were subjected to the same pretreatment and analysis procedures as the studied samples. All the results of quality control analysis were in the reference range provided by the manufacturer of the certified materials (Table 3).

The recovery figures for the analytical methods used in estimation of Ca, Mg, Fe, Zn, Cu, Mn, and Se were 101%, 98.7%, 104%, 101%, 100.5%, 98.5%, and 98%, respectively. The precision values of the methods for determination of Ca, Mg, Fe, Zn, Cu, Mn, and Se were 3.4%, 3.6%, 2.6%, 2.4%, 2.2%, 2.8%, and 3.8%, respectively. 

### 2.5. Statistical Analysis

Statistical analysis was performed using Statistica software 13.0 (Statsoft, Kraków, Poland). The normality of the data was verified by means of the Shapiro–Wilk test and the Kolgomorov–Smirnov test. No criterion of normality was observed, therefore, to calculate significant differences, the Kruskal–Wallis and Mann–Whitney U tests were applied. Differences were considered significant when *p* < 0.05.

### 2.6. Assessment of Mineral Content in the Dried Mushrooms in Relation to Nutrition Standards

To assess the fulfillment of the demand for studied trace elements as a result of the consumption of a portion of dried mushrooms by an adult, nutrition standards for daily reference intake (DRI) were used. The DRI for individual elements is as follows: Ca⁠—800 mg/day, Mg—375 mg/day, Fe—14 mg/day, Zn—10 mg/day, Cu—1 mg/day, Mn—2 mg/day, and Se⁠—55 µg/day. A food product can be considered to be a source of a mineral provided that it contains at least a significant amount of this nutrient, defined as a minimum of 15% DRI in a standard portion [28]. The standard portion of fresh mushrooms consumed per person in Poland is 100 g and dry mass accounts for about 10% of mushrooms’ weight [29,30]. We assumed that an average person weighed 70 kg and that a standard portion of dried mushrooms consumed was 10 g. According to published data, the average daily consumption of mushrooms by a person weighing 70 kg is estimated as 27 g/day for fresh mushrooms, which means 2.7 g/day in the case of dried mushrooms [30]. The content of the analyzed elements was compared with Polish nutrition norms for these elements at the level of the daily reference intake (DRI) for an adult [28]. 

## 3. Results

### 3.1. The Concentrations of the Assessed Trace Elements in Dried Mushrooms

The concentrations of Ca, Mg, Fe, Zn, Cu, Mn, and Se in dried mushrooms are presented in Table 4.

Analysis of the results indicates that the content of trace elements varies, depending on the species of studied mushrooms. In the case of *X. badius*, the comparative content of the analyzed elements can be represented as follows: Mg > Zn > Fe > Ca > Cu > Mn > Se. The concentrations of trace elements in *B. edulis* were different: Mg > Fe > Zn > Ca > Cu > Mn > Se.

In *B. edulis*, the median Ca concentration was higher (79.9 mg/kg) than that in *X. badius* (56.8 mg/kg). The difference in Ca concentration between studied mushroom species was statistically significant (*p* < 0.01). The maximum quantities of Ca found in dried *B. edulis* and *X. badius* were 154.8 mg/kg and 131.0 mg/kg, respectively.

In *X. badius*, the median Mg concentration amounted to 1024.6 mg/kg. On the other hand, the concentration of Mg in *B. edulis* was lower (981 mg/kg). The maximum concentration of Mg found in *X. badius* was 1422.3 mg/kg. The demonstrated increase in the concentration of Mg in *X. badius* was statistically significant in comparison with that recorded for *B. edulis* (*p* < 0.001).

The median concentration of Fe in *X. badius* was 80.9 mg/kg, i.e., 2.7 times lower than Fe level found in *B. edulis* (218.5 mg/kg). The maximum value of Fe found in *B. edulis* reached even 707.1 mg/kg. The difference in Fe content between these two species of mushrooms was statistically significant (*p* < 0.00001).

In *B. edulis*, the median Zn concentration was 93.5 mg/kg. In *X. badius*, the amount of this element was much higher: 174.2 mg/kg; the maximum value detected being as much as 365.6 mg/kg. This means that the median Zn concentration in *X. badius* was almost 186% of that found in the other species. The difference was statistically significant (*p* < 0.000001).

The median Cu concentration in *B. edulis* was 22.2 mg/kg. In comparison, its content in *X. badius* proved to be nearly 150% higher (median concentration 33.1 mg/kg). The difference was statistically significant (*p* < 0.000001). The maximum quantity of Cu in *B. edulis* equaled 72.2 mg/kg.

The Zn:Cu ratio was significantly higher in *X. badius* than in *B. edulis*. In the former species, it was 5.9, with the minimum value of 3.6 and the maximum value of 10.4. All values were lower in *B. edulis* (mean: 4.2, minimum: 2.0, maximum: 6.7)

The median Mn concentration in *B. edulis* was 22.5 mg/kg and 20.6 mg/kg in *X. badius.* The difference was statistically significant (*p* < 0.05). The maximum values of Mn were 36.6 mg/kg in *B. edulis* and 31.5 mg/kg in the other mushroom species. 

The median Se concentration in *B. edulis* was 6013.1 µg/kg and it was more than 38 times higher than the amount of this element in *X. badius* (156.2 µg/kg). The difference in terms of Se content between these two species of studied mushrooms was statistically significant (*p* < 0.000001). In some samples, Se content was as high as 17,265.8 µg/kg.

### 3.2. Assessment of Daily Reference Intake (DRI) Coverage for the Tested Elements after the Consumption of a Standard Portion (2.7 g) of Dried Mushrooms

The concentrations of the studied elements assessed in a 2.7 g portion of dried mushrooms are presented in Table 5. 

**Figure 2:**
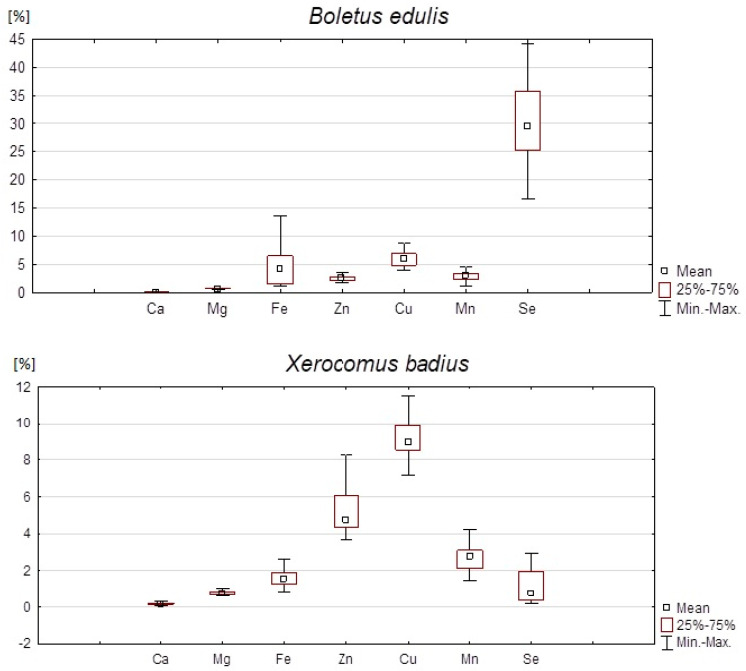
Percentage of DRI of Ca, Mg, Fe, Zn, Cu, Mn, and Se after consumption of 2.7 g of dried mushrooms.

The percentages of the daily reference intake of the studied elements assessed in a 2.7 g portion of dried mushrooms are shown in Figure 2.

We found that, of the two studied dried wild-growing mushrooms, *B. edulis* was the better source of Se. A portion (2.7 g) of dried *B. edulis* covered almost 32% of the DRI for Se, with the maximum value of nearly 85%. In comparison, a standard portion of *X. badius* provided only 1.4% of the reference value for Se (maximum 6.2%). Similarly, consuming 2.7 g of dried *B. edulis* ensured a higher percentage of DRI for Mn and Fe after. We determined that a standard portion of this mushroom species fulfilled 2.99% of the DRI for Mn (maximum value: nearly 5%) and 4.5% of the DRI for Fe (maximum: 13.6%). Meanwhile, a portion of *X. badius* provided only 2.67% and 1.69% of DRI for Mn and Fe, respectively. Both studied mushroom species are a modest source of Ca. A standard portion of dried *B. edulis* constitutes only 0.03% and *X. badius* 0.02% of the DRI for this element (Figure 2).

On the other hand, dried *X. badius* is richer in Zn, Mg, and Cu than *B. edulis*. Consuming of 2.7 g of *X. badius* ensures more than 5% of the RI for Zn, with the maximum value near 10%. In comparison, the same portion of *B. edulis* provides only 2.6% of the reference value for Zn (maximum 4.4%). As regards to Mg, the difference between the two species is smaller. A standard portion of dried mushrooms covers 0.76% and 0.69% of DRI for *X. badius* and *B. edulis*, respectively. On the other hand, we estimate that the mean percentage of DRI for Cu constitutes more than 9% for *X. badius* and 6.8% for *B. edulis*. Moreover, we found that the maximum DRI for Cu is higher in *B. edulis* (19.5%) than in *X. badius* (11.5%) (Figure 2).

## 4. Discussion

Mushrooms are a valuable product, richer in some elements than most vegetables. They are able to store minerals in large quantities, and the uptake of trace elements considered physiologically essential in mushrooms is species-dependent [31]. It is worth investigating whether edible dried mushrooms might be a source of mineral components that are indispensable for the functioning of the human organism. Therefore, we focused on the quantification of the elements present in the two most popular mushroom species, *B. edulis* and *X. badius,* and evaluated their contribution to the recommended intake of elements. 

*B. edulis* is the most frequently studied mushroom species. In our study, we found that the concentration of Ca in *B. edulis* was 79.9 mg/kg and 56.8 mg/kg in *X. badius*. The results we obtained are close to those of Gałgowska and Pietrzak-Fiećko, who detected 75.3 mg/kg of Ca in *B. edulis* [32]. However, the content of Ca in *B. edulis* in our study was lower than that recorded by Brzezicha-Cirocka et al., (110–300 mg/kg) [33].

In *X. badius*, we found 1024 mg/kg of Mg, and in *B. edulis* we found 981.5 mg/kg, which is higher than what is reported in the literature. In mushrooms from China, the content of this element amounted to 574–708 mg/kg, and in samples from Africa 540–860 mg/kg [34,35]. Brzezicha-Cirocka et al., detected 850–910 mg/kg of Mg in *B. edulis* collected in Tarnobrzeska Plain and Morąg [33]. 

We found that the median Fe concentration in *X. badius* was 80.9 mg/kg but in *B. edulis,* it was much higher: 218.5 mg/kg. Our results are consistent with the findings by Brzezicha-Cirocka et al., who detected 210 mg/kg of Fe in *B. edulis* from the Tarnobrzeg Plain [33]. Other researchers found as much as 9685 mg/kg of Fe in mushrooms picked in a forest located near a highway in Turkey [36]. This area had been exposed to traffic pollution for many years and it is commonly known that mushrooms manifest a high capacity to absorb elements from the soil. The mean concentration of Fe in *X. badius* recorded by Kuziemska et al., was 109 mg/kg, which is higher than our results in this mushrooms species [37]. We also established that the standard portion of these mushrooms fulfilled 4.5% of the DRI for Fe (maximum 13.6%) and these findings are consistent with the literature data (6.79–12.2%) [32].

Zn concentrations in mushrooms have been analyzed in many publications. Most often, they fell into the range of 50–150 mg/kg [38]. We found that the median Zn concentration was close to 93.5 mg/kg in *B. edulis* and over 174 mg/kg in *X. badius*. In Giannaccini et al., Zn content in *B. edulis* exceeded 120 mg/kg but Mleczek et al., detected quantities of 72–88 mg/kg in uncontaminated areas, which is consistent with the data obtained in this study [39,40]. Research conducted by Kuziemska et al., in *X. badius* showed a much lower Zn concentration (60.6 mg/kg) than that established in our study [37]. However, some mushroom species have a strong ability to accumulate Zn, an example of which could be *Russula atropurpurea.* This mushroom, harvested in unpolluted areas in the Czech Republic and Slovakia, contained as much Zn as 1062 mg/kg. The explanation for this is the presence of functional peptides in sporocarps, which bind this element [41]. On the other hand, some authors claimed that *B. edulis* covered 13.3–18.2% of RDA for Zn, far more than what we obtained in our study [42]. Consumption of a standard portion of *X. badius* covered more than 5% of the DRI for Zn (maximum value: nearly 10%), whereas *B. edulis* provided only 2.6% of the reference amount of Zn (maximum 4.4%). Even higher values were obtained by other researchers, who claimed that *B. edulis* provided 35.8–49.2% of the Daily Demand Coverage (DDC) [32].

Data from the literature show that the average Cu content in mushrooms is usually 100–300 mg/kg [38]. We detected much lower concentrations of the element in dried mushrooms. The median content of copper in *B. edulis* was 22.2 mg/kg, while in *X. badius*, it was 33.1 mg/kg. Our findings are similar to those obtained by Karmańska and Wędzisz: 22.0–22.9 mg/kg [43]. Moreover, Brzezicha-Cirocka et al., found 15–70 and 6–72 mg/kg of Cu in *B. edulis* picked in Morąg and on the Tarnobrzeska Plain, respectively [33]. Our results are also consistent with Kuziemska et al., who examined the content of Cu in *X. badius* and found that it amounted to 17.1–38.4 mg/kg [37]. Cu content may be elevated when mushrooms absorb this element from a contaminated area. Norwegian authors reported that the concentration of Cu measured in *B. edulis* that had been growing around a copper smelter was 427 mg/kg [44]. Moreover, we also found that the maximum DRI for Cu was higher for *B. edulis* (19.5%) than *X. badius* (11.5%). Our results are consistent with Mirończuk-Chodakowska et al., who reported that a portion of *B. edulis* covered 18.1% of the Recommended Dietary Allowance (RDA) [42].

We concluded that the Zn:Cu ratio was significantly higher in *X. badius* (5.9) than in *B. edulis* (4.2). Zn and Cu are microelements that compete for the binding places of some transporters and metal-binding proteins and a disproportion of Zn:Cu in the human body may have an effect on oxidative stress [45]. Decreased concentration of Zn and increased content of Cu affects the antioxidant properties of multiple enzymes such as superoxide dismutase (SOD) [46]. Therefore, it is especially important to maintain the correct Zn:Cu ratio in food products, which will translate into the maintenance of the accurate ratio in the body, and thus its proper functioning. Our research shows that the Zn:Cu ratio in mushrooms is favorable, higher than that found in legumes or cottage cheeses. Górska-Warsewicz et al., demonstrated that the content of Zn in cottage cheeses was approximately 3.7-fold higher than that of Cu. Margier et al., found that Zn content in legumes was 2.7–4.1-fold higher in relation to Cu [47,48]. The Zn:Cu ratio in the studied dried *X. badius* was comparable to that in cereals (5.2–6.4) [49].

Brzezicha-Cirocka et al., found that Mn content in *B. edulis* fell between 9 and 47 mg/kg [33]. This is in line with our study, where the concentration of manganese in *B. edulis* proved to be 22.5 mg/kg. In *X. badius,* we detected 20.6 mg/kg of Mn, i.e., nearly 10 times more than Kuziemska et al. [37]. We determined that a standard portion of *B. edulis* covered 2.98% of the DRI for Mn (maximum value: nearly 5%)—less than reported by Gałkowska and Pietrzak-Fiećko, who claimed that a portion of *B. edulis* covered from 12.3 to 15.7% of the daily requirement for Mn [32].

In the present study, Se content in *B. edulis* was 6013.1 µg/kg, much higher than the concentration of this element in *X. badius* (156.2 µg/kg). Mirończuk-Chodakowska et al., recorded more than 2 times higher Se concentrations in *B. edulis*, reaching 13,300 µg/kg, while Falandysz found approximately 20,000 µg/kg of Se in these mushrooms [42,50]. It is commonly known that the content of Se in food is closely related to its content in the environment, which can explain the above differences [51]. We found that *B. edulis* was the better source of Se because consuming a portion of dried *B. edulis* covered almost 32% of the DRI for Se, with the maximum value of almost 85%. Mirończuk-Chodakowska et al., reported that consuming a 100 g portion of fresh mushrooms by adults covered approximately 211% of the daily requirement for Se [42]. Our results suggest that Se content in a standard portion of dried *B. edulis* constitutes a significant amount because it accounts for more than 15% of the daily reference intake [28]. Therefore, according to the Commission Regulation (EU) No. 432/2012 of 16 May 2012 (Journal of Laws UE No. 136/1 of 25 May 2012, as amended), the following nutritional claims approved by the European Food Safety Authority (EFSA) may be used for selenium; it contributes to normal spermatogenesis, the maintenance of normal hair and nails, the normal function of the immune system, and the normal thyroid function, as well as the protection of cells from oxidative stress [52].

It is also worth paying attention to the issue of labeling dried mushrooms in a way that will not mislead consumers about the origin and nutrition declaration of the product. According to Article 26 (2)(a) of the Regulation No. 1169/2011, “Indication of the country of origin or place of provenance shall be mandatory where failure to indicate this might mislead the consumer as to the true country of origin or place of provenance of the food, in particular if the information accompanying the food or the label as a whole would otherwise imply that the food has a different country of origin or place of provenance” [28]. The judgment of the Court of Justice of the European Union in case C-485/18 Groupe Lactalis v. Premier ministre et al., noted that harmonization does not prevent Member States from adopting legislation requiring certain additional particulars of origin provided that the requirements laid down in Regulation No. 1169/2011 are met [53]. While purchasing the mushrooms used in this study, we were not always able to identify the country or place of origin of the products. This means that there are still problems with the implementation of this obligation. Regarding the nutritional value, it should be noted that according to Article 30 (1) of Regulation No. 1169/2011, product packaging must display: (a) energy value, and (b) the amounts of fat, saturates, carbohydrate, sugars, protein, and salt. This minimum requirement is generally met. Information on vitamins and minerals is not mandatory, and it was not given on the packaging of the mushrooms we bought. Article 30(2) provides that “The content of the mandatory nutrition declaration referred to in paragraph 1 may be supplemented with an indication of the amounts of one or more of the following: (a) mono-unsaturates; (b) polyunsaturates; (c) polyols; (d) starch; (e) fibre; (f) any of the vitamins or minerals listed in point 1 of Part A of Annex XIII, and present in significant amounts as defined in point 2 of Part A of Annex XIII.” Among these vitamins and minerals are listed the elements studied by us; their DRIs are discussed in Section 2.6 of this article. On the basis of the conducted research, it can be concluded that, apart from Ca, all the other elements meet the criterion provided by the regulation and can (and in our opinion, should) be indicated on product labels.

## 5. Conclusions

The studied mushroom species have varying contents of the tested elements. The highest values of Se, Fe, Mn, and Ca were found in *B. edulis*, but *X. badius* was characterized by higher contents of Zn, Mg, and Cu. Among the studied mushrooms, *B. edulis* can be regarded as a nutritional source of Se. As far as the other microelements are concerned, the tested mushrooms can be treated as additional enrichment of the diet. However, the above species of dried mushrooms are not a source of the studied macroelements. In addition to Ca, the elements tested can (and should), in the light of current legislation, be indicated on the labels of products, which may help increase consumer awareness of the nutritional value of mushrooms.

## Figures and Tables

**Table 1:** Characteristics of studied samples of mushrooms.

Supermarket/City	Company	*X. badius* No. of Batches	*B. edulis* No. of Batches
Auchan	PolGrzyb	5	4
Auchan	Tagros Polska	5	6
Kaufland	JamPol	10	10
Lidl	Nasza Chata	10	10
Carrefour	RunoPol	5	5
Tesco	RunoPol	5	5

**Table 2:** The analytical conditions of electrothermal AAS technique (Se) and flame AAS technique (Ca, Mg, Fe, Zn, Cu, and Mn) in determining the content of elements in dried mushrooms.

Element	Wavelength (nm)	Lamp Current (mA)	Graphite Cuvette—Temperature Conditions
Ca	422.7	7.5	-
Mg	285.2	7.5	-
Fe	248.3	12.5	-
Zn	213.9	6.5	-
Cu	324.8	7.5	-
Mn	279.5	7.5	-
Se	196.0	14.5	Drying 70/100 °C Ashing 600/600 °C Atomization 2700/2700 °C Cuvette cleaning 2800/2800 °C

Ca—calcium; Mg—magnesium; Fe—iron; Zn—zinc; Cu—copper; Mn—manganese; Se—selenium.

**Table 3:** Certified reference materials used in checking accuracy of methods used for determining the content of elements in dried mushrooms.

Element	Reference Material	
	Dried Mushroom Powder	Simulated Diet D
	Certified Levels of Elements ± Expanded Uncertainty (mg/kg Dry Mass)	
Ca	-	510 ± 37
Fe	-	199 ± 16
Mg	-	676 ± 65
Zn	113.30 ± 3.28	-
Cu	18.73 ± 0.70	-
Mn	-	10.2 ± 0.6
Se	17.43 ± 1.36	-

Ca—calcium; Mg—magnesium; Fe—iron; Zn—zinc; Cu—copper; Mn—manganese; Se—selenium.

**Table 4:** Concentrations of Ca, Mg, Fe, Zn, Cu, Mn (mg/kg), and Se (µg/kg) in dried mushrooms.

	Element						
Mushroom Species	Mean Content	Median	Min.	Max.	SD	Q_1_	Q_3_
	Ca						
*B. edulis* (n = 40)	82.1	79.9	18.7	154.8	31.0	63.5	106.6
*X. badius* (n = 40)	67.5	56.8	21.7	131.0	27.6	48.5	76.7
*B. edulis* vs. *X. badius*	*p* < 0.01						
	Mg						
*B. edulis* (n = 40)	964.1	981.5	791.3	1143.3	86.2	894.7	1031.0
*X. badius* (n = 40)	1060.2	1024.6	868.6	1422.3	128.2	951.1	1158.6
*B. edulis* vs. *X. badius*	*p* < 0.001						
	Fe						
*B. edulis* (n = 40)	233.4	218.5	56.3	707.1	163.2	78.1	339.5
*X. badius* (n = 40)	87.8	80.9	42.9	219.9	33.3	67.0	97.2
*B. edulis* vs. *X. badius*	*p* < 0.00001						
	Zn						
*B. edulis* (n = 40)	97.9	93.5	63.8	163.6	25.7	77.5	104.4
*X. badius* (n = 40)	197.2	174.2	136.6	365.6	55.7	161.1	224.0
*B. edulis* vs. *X. badius*	*p* < 0.000001						
	Cu						
*B. edulis* (n = 40)	25.8	22.2	14.6	72.2	11.9	18.1	25.6
*X. badius* (n = 40)	33.9	33.1	22.4	42.6	4.5	31.6	36.6
*B. edulis* vs. *X. badius*	*p* < 0.000001						
	Zn:Cu ratio						
*B. edulis* (n = 40)	4.2	4.1	2.0	6.7	1.1	3.6	4.5
*X. badius* (n = 40)	5.9	5.4	3.6	10.4	1.7	4.7	7.0
*B. edulis* vs. *X. badius*	*p* < 0.000001						
	Mn						
*B. edulis* (n = 40)	22.1	22.5	9.0	36.6	6.0	17.9	24.8
*X. badius* (n = 40)	19.8	20.6	10.7	31.5	4.6	15.9	22.9
*B. edulis* vs. *X. badius*	*p* < 0.05						
	Se						
*B. edulis* (n = 40)	6501.6	6013.1	3391.1	17,265.8	2297.7	5138.6	7295.0
*X. badius* (n = 40)	282.4	156.2	41.1	1262.7	308.7	68.8	405.1
*B. edulis* vs. *X. badius*	*p* < 0.000001						

Ca—calcium; Mg—magnesium; Fe—iron; Zn—zinc; Cu—copper; Mn—manganese; Se—selenium; n—number of samples; SD—standard deviation; Q1—lower quartile; Q3—upper quartile; *p* < 0.05—statistically significant value.

**Table 5:** Content of Ca, Mg, Fe, Zn, Cu, Mn (mg/kg), and Se (µg/kg) in a standard portion (2.7 g) of dried mushrooms.

Element				
Mushroom Species	Mean Content	Min.	Max.	SD
Ca				
*B. edulis*	0.22	0.05	0.42	0.08
*X. badius*	0.18	0.06	0.35	0.07
Mg				
*B. edulis*	2.60	2.14	3.09	0.23
*X. badius*	2.86	2.35	3.84	0.35
Fe				
*B. edulis*	0.63	0.15	1.91	0.44
*X. badius*	0.24	0.12	0.59	0.09
Zn				
*B. edulis*	0.26	0.17	0.44	0.07
*X. badius*	0.53	0.37	0.99	0.15
Cu				
*B. edulis*	0.07	0.04	0.20	0.03
*X. badius*	0.09	0.06	0.12	0.01
Mn				
*B. edulis*	0.06	0.02	0.10	0.02
*X. badius*	0.05	0.03	0.08	0.01
Se				
*B. edulis*	17.55	9.16	46.62	6.20
*X. badius*	0.76	0.11	3.41	0.83

Ca—calcium; Mg—magnesium; Fe—iron; Zn—zinc; Cu—copper; Mn—manganese; Se—selenium; SD—standard deviation.

## Data Availability

The data presented in this study are available upon request from the corresponding author.
